# Evaluating care farming as a means to care for those in trauma and grief

**DOI:** 10.1016/j.healthplace.2019.102281

**Published:** 2020-03

**Authors:** Joanne Cacciatore, Richard Gorman, Kara Thieleman

**Affiliations:** a School of Social Work, Arizona State University, United States; b Department of Geography, University of Exeter, United Kingdom; c School of Social Work, Faculty Associate, Arizona State University, United States

## Abstract

The interrelationships between nature, health, and wellbeing are increasingly recognized and incorporated into therapeutic interventions. Care farming, the concept of utilizing agricultural places and practices for providing care, therapy, and rehabilitation, is a paradigmatic example of this shift. This mixed method study empirically evaluates the efficacy of care farming as an intervention for individuals affected by traumatic grief, a complex experiential condition. Both quantitative and qualitative results suggest this care farm intervention was beneficial, yielding significant reductions in subjective distress to grief intensity. The study's findings add to the growing body of evidence on care farming and support green care as a therapeutic potential for individuals affected by traumatic grief.

## Highlights

• Few interventions have been successfully tested with those suffering traumatic grief. Care farming is a potential alternative means toward provision of care for the traumatically grieving population.
• After a two-day, 10 h intervention, participants experienced significantly less subjective trauma and distress in pre/post tests using the TGI psychometric measures.
• Rich qualitative data reveal that participants felt supported, connected, and yielded therapeutic benefit.
• Given the lack of consensus on the use psychotropic medications for grief, green models, such as care farming, provide to care that is not limited to a medical model.

## 1 Introduction

The last two decades have seen a proliferation of therapeutic interventions broadly categorised under the framework of “green care.” The green care paradigm includes a broad and heterogeneous variety of models, activities, and practices, often highly differentiated in application by geography and culture. However, this diversity can be conceptualised as having “as their central dimension the engagement of the individual with nature (in a structured and facilitated way) to provide a benefit to health” (Sempik, 2008, p. 223). The links between nature, health, and wellbeing are well reported (Hartig et al., 2014; Maller et al., 2006).

Recent scholarship has demonstrated an interest in the interfaces between grief and green-care type interventions (Gorman and Cacciatore, 2017; Lin et al., 2014; Machado and Swank, 2018; Symington, 2012). Individuals exposed to deaths that are sudden and unexpected, violent, or involve the death of a child can be described as experiencing “traumatic bereavement” (Thieleman and Cacciatore, 2014). Traumatic grief can put individuals at risk of long-term emotional, mental, and physical health impairments and adverse health behaviours (Prigerson et al., 1997). How to assist traumatically bereaved groups is an ongoing question, entangled with complex questions of how grief is understood and imagined (Thieleman and Cacciatore, 2014).

The purpose of this mixed methods study is to empirically investigate the efficacy of a particular form of green care, care farming, as an intervention for individuals affected by traumatic grief, drawing on both quantitative and qualitative approaches. The conceptual contexts which framed this study are introduced, drawing on the interdisciplinary literature that discusses ideas of “therapeutic landscapes” and “therapeutic communities,” before contextualising “care farming” and how this intervention might be applied to the interface of trauma and grief.

## 2 Therapeutic landscapes and communities

Care farms have been described as both “therapeutic landscapes” (Gorman, 2017a; Kaley et al., 2019; Leck et al., 2014) and “therapeutic communities” (Elings and Hassink, 2008; Haigh, 2012; Loue et al., 2014). This study draws on these two conceptual frameworks to demonstrate the ways in which care farming can produce therapeutic experiences through mutually reinforcing processes and experiences of “place-as-setting” and “place-as-group-dynamic” (Moon et al., 2006).

### 2.1 Therapeutic landscapes and communities

The conceptual framework of “therapeutic landscapes” (Gesler, 1992) has been used to explore how people's experience of place can impact and (re)shape experiences of health and wellbeing. The concept adopts a socio-cultural framework to draw attention to “the complex intermingling of physical, social and symbolic processes that determine a place's potential to positively or negatively affect health” (Kaley et al., 2019, p. 10). The therapeutic landscapes concept holds great potential in furthering understandings of the sociology of green care (Sempik et al., 2010).

The framework draws attention to the very geography of therapeutic interventions and communities, focusing on place as active and constitutive of health, while recognizing that it is not possible to separate experiences of health and wellbeing from the places in which they are experienced (Kearns, 1993). Interventions that promote health must also attend to the confluence of environmental, interpersonal, and individual influences in therapeutic landscapes where the “social and spatial are intimately intertwined” (Gesler, 1992, p. 744).

Many of the ideas associated with the therapeutic landscape concept are also reflected in the therapeutic community concept (Gesler, 1993). Therapeutic communities are group-based treatment programs, many of which use farms or gardens as a focus within their work (Sempik et al., 2010). While therapeutic landscapes tend to be associated with the healing properties of the physical or built environment, “therapeutic communities operate on a more sociological level in which place-as-setting may matter less than place-as-group-dynamic” (Moon et al., 2006, p. 143).

Though having a long history (see Hickman, 2013), present day ideas of therapeutic communities stem from the treatment of psychological casualties during World War II in Britain using a novel and non-hierarchical approach to treating combat-related trauma (Fussinger, 2011; Perfas, 2003) and “an attempt to replace the prevailing extremely poor conditions and primarily custodial care in mental hospitals with truly therapeutic environments and treatments” (Gesler, 1993, p. 176). Modern therapeutic community models are more adaptable, used in both residential and non-residential settings. Gesler (1993) identified a series of guiding principles of therapeutic communities: a holistic view of patient treatment, the fostering of a spirit of community, a sense of permissiveness, a democratic environment within which patients participate actively in treatments, the inclusion of a wide variety of experiences and relationships as part of treatment, and a goal to prepare patients to take up social roles outside the therapeutic community. Therapeutic communities aim to encompass a holistic notion of health, drawing on social relations, a sense of place, and everyday activities. Gesler (1993) noted that these principles are often difficult to put into practice, particularly in the face of challenges by “the biomedical establishment” (p. 177), with many therapeutic communities operating in opposition to what they view as the pathologization, over-zealous medicalization, and expropriation of health (Sempik et al., 2010).

## 3 Care farming

Care farming, the concept of combining agricultural places and practices with the provision of health, social, and educational services, has arisen as an innovative modality for providing forms of care, therapy, and rehabilitation (Hassink et al., 2010). As mentioned, scholars have drawn on both ideas of therapeutic landscapes (Gorman, 2017a; Kaley et al., 2019; Leck et al., 2014) and therapeutic communities (Elings and Hassink, 2008; Haigh, 2012; Loue et al., 2014) to explore the processes and practices through which care farms can produce therapeutic experiences.

Care farming has been defined as “the use of commercial farms and agricultural landscapes as a base for promoting mental and physical health through normal farming activity” (Hine et al., 2008, p. 247). The extent of both “farming” and “care” varies on a case by case basis, with the “care” being delivered through a combination of therapeutic contact with farm animals (often involving providing care to the animals), horticultural activities, social interaction, and wider nature-based activities (Kraftl, 2014). All of this occurs within the specific place-based context of an agricultural, and often rural, landscape, which influences expectations and experiences (Gorman, 2020). As Kaley et al. (2019, p. 11) noted, care farming is “increasingly being advocated as a viable alternative to more traditional forms of health and social care.”

Participation in care farming has been shown to have a wide variety of benefits, including decreasing anxiety, stress (Leck et al., 2015), and depressive symptoms (Pedersen et al., 2012) as well as increasing self-esteem (Hine et al., 2008), self-efficacy (Kogstad et al., 2014), social interaction (Iancu et al., 2014), and psychological wellbeing (Ellingsen-Dalskau et al., 2016). These benefits emerge through the relationships that develop on care farms and mechanisms that enable individuals to derive therapeutic effect, such as meaningful work (Hassink et al., 2010; Iancu et al., 2014; Kogstad et al., 2014), social interaction (Elings and Hassink, 2008; Hassink et al., 2010; Pedersen et al., 2012), and encounters with animals (Gorman, 2017b; Hassink et al., 2017; Leck et al., 2014).

Human-animal relationships play a significant role in the care farm experience, with Hassink et al. (2017: 8) describing animals as being ‘the fabric of the care farm’. Indeed, there is a wide body of literature which has catalogued the health benefits of human-animal interactions, leading Beck and Katcher (2003: 87) to summarize that ‘there is solid evidence that animal contact has significant health benefits and that it positively influences transient physiological states, morale, and feelings of self-worth’ (p. 87). However, Hassink et al. (2017: 3) also note that ‘the role and effect of farm animals at care farms for different client groups is a relatively new area of research that requires further study’. Our work here thus moves to consider the connection to animals as an important element of the program and consider how the presence of animals is valued by participants experiencing traumatic grief.

Care farming has been widely used for a variety of populations and conditions. A novel community-based care farm has been established that aims to specifically help those experiencing traumatic grief. This study explores the effects of this care farm on study participants.

## 4 Trauma and grief

Grief that results from the death of a loved one under traumatic circumstances, particularly when the relationship is highly dependent, may be experienced differently from grief related to more natural and expected deaths (Adler, 1943; Jacobs, 1999). Homicide, suicide, violent, disfiguring (a person's unrecognizability) or sudden deaths, and the deaths of children seem to evoke intense and enduring emotional distress in parents and families that does not abate merely with the passage of time (Cacciatore et al., 2014).

Traumatic grief is a complex experiential condition, having biological, psychological, social, and cultural facets. It can incite a long-term and intense form of distress (Cacciatore, 2007), putting individuals at risk of a variety of emotional, mental, and physical health impairments and adverse health behaviours (Prigerson et al., 1997), alongside wider impacts affecting income, employment status, and relationships (van den Berg et al., 2017). For instance, the death of a child family member, in short-term costs, is approximately $22,000 per family in the first year, conservatively (Fox et al., 2014). van den Berg et al. found that “parents who lose a child end up on a lower long-term income trajectory,” and those negative effects persist long after the death (2017, p.17).

Attempts to grapple with the complex intersection of trauma and grief have seen an increasing move to “medicalize” those affected with the creation of Persistent Complex Bereavement Disorder in the fifth edition of the Diagnostic and Statistical Manual of Mental Disorders. This move has been celebrated for capturing grief-related problems unrelated to depressive or posttraumatic stress symptoms (Bonanno et al., 2007), but critiqued for introducing the risk of pathologizing normal responses to loss (Wakefield, 2012) and advocating normative symptoms and arbitrary timelines on grief that may invalidate and silence the wide range of normal experiences related to traumatic bereavement (Thieleman and Cacciatore, 2014). As such, how to best support traumatically bereaved groups is an ongoing and complex question.

## 5 Methods

Our methodology follows a mixed methods approach in order to ‘gain a better to gain a better understanding of the connections or contradictions between qualitative and quantitative data’ (Shorten and Smith, 2017: 75). Mixed methods approaches are increasingly used within healthcare research, as they allow the exploration of ‘diverse perspectives and uncover relationships that exist between the intricate layers of our multifaceted research questions’ (Shorten and Smith, 2017: 75). Research associated with ‘therapeutic landscapes’ tends to be qualitative, whilst research exploring a person's experiences of traumatic grief leans toward quantitative. Mixing these approaches allows us to gain greater insight, but also speak with legitimacy to multiple audiences across different disciplines. In this section, we briefly introduce our methods of recruitment, the setting where the study took place, the specific contexts of the intervention being studied, and our quantitative and qualitative methods of data collection and analysis.

### 5.1 Recruitment

Following Institutional Review Board approval via Arizona State University, recruitment materials were published for two weeks on the social media sites of several non-governmental agencies that help families facing traumatic grief. Prospective adult participants were invited to take part in a study whose stated intention was to understand the effects of a care farm intervention on their subjective experience of trauma and grief. They were offered a web link for registration and initial intake, this included a section obtaining participant's informed consent to take part in the research. Eligible participants were then contacted to schedule the in-person intervention. Given the potential for sensitive topics to be discussed during the course of participants' involvement in research, a list of nonprofits specifically aimed at supporting grieving individuals and families was provided to participants as well as a resource brochure on grief.

One to two weeks prior to arrival at the care farm, respondents provided demographic and loss-related information and completed a standardized traumatic grief measure, chosen to quantify the subjective intensity of their psychological state both pre and post intervention. Two to five weeks after the intervention, they completed the measure again, and two to five weeks after that, they were invited to participate in a qualitative, semi-structured interview with a research team member who was not involved with the delivery of the intervention. At each of these stages, informed consent was obtained from participants, that they were comfortable taking part in the research.

### 5.2 Setting

The carefarm^1^ where the intervention took place is located on ten acres in Northern Arizona. It is a new and novel program started in 2017 by an international non-governmental organization devoted to helping families coping with traumatic grief. Bereaved parents are the most frequent clients, followed by siblings, spouses/partners, and those grieving the traumatic or early death of a parent. This carefarm only takes in animals rescued from abuse, neglect, and homelessness. There were 30 animals on the carefarm during the intervention including horses, donkeys, pigs, sheep, goats, dogs, and cats. All the animals at the carefarm exist in an egalitarian model; that is, the farm aims to provide a level of autonomy to the animals, with their willingness to interact with humans, or not, being respected and foregrounded. No animal is ever haltered or coerced into interacting with people. Participants were encouraged to build a relationship of trust with the animals that unfolded in whatever way was comfortable and desirable for both them and the animals.

There are several formally designated “restorative spaces” that together constitute the therapeutic landscape of the carefarm including “The Quiet Place,” a small grotto under a large Ash tree by a waterway where families paint stones and leave them in the rock wall; a heavily treed river area with swings, a firepit, and kayaks; a gazebo set in the middle of a small pasture; and various spaces for equines who, unlike the other animals who roam the entire carefarm freely, graze in enclosed pastures for safety. Though the carefarm is secularized, the guiding principle is unitive “ahimsa,” or dynamic compassion, the foundation upon which many spiritual traditions are built (Altman, 2017). The concept of active compassion toward all who suffer begins with humans and extends to non-human species and the earth. Thus, this care farm is sustainable and vegan, requiring guests to adhere to these principles while on the premises.

### 5.3 Intervention

**Graph 1:**
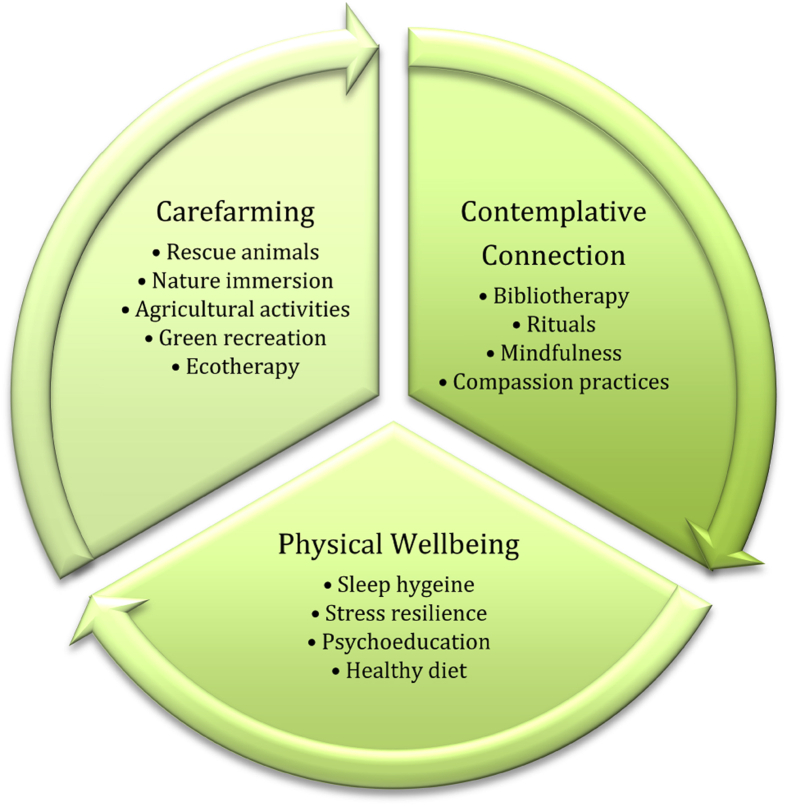
Green Care Model for Traumatic Grief.

The intervention lasted for 10 h over the course of two consecutive days utilizing a model of green care (Cacciatore and Gorman, 2016) endorsed by the nongovernmental agency that runs the carefarm (See Graph 1). Only those who suffered the traumatic death of a child or sibling participated. Clients came to the carefarm individually, one at a time or, if partnered, as a couple and were scheduled based on availability over the course of four months. They received traumatic grief-focused counseling with one of three trained providers. Reflexivity in attending to each client's needs is a cornerstone of this model, however, all clients received between 4 and 6 hours of counseling over two days. Participants' remaining time was spent exploring the nature spaces on the carefarm, interacting with or caring for the animals, listening to the animals' stories as conveyed by their counselor, and engaged in various rituals such as painting a memorial rock or creating a metal medallion to hang on the remembrance tree. There were no differences in the siblings' intervention compared to the parents' intervention. When the intervention ended, clients returned to their temporary housing until the next day.

### 5.4 Data collection and analysis

**Traumatic Grief Inventory Self-Report.** The Traumatic Grief Inventory Self-Report (TGI-SR) is commonly used in research with this population to assess a person's experiences of traumatic grief. The TGI-SR is an 18-item measure reflecting the criteria for both Persistent Complex Bereavement Disorder (PCBD) and Prolonged Grief Disorder (PGD), a related proposed disorder (Boelen and Smid, 2017). Items are scored on a 5-point scale, ranging from 1 = “never” to 5 = “always; ” a total score is calculated by summing the items. Higher scores reflect higher grief intensity and a cutoff of 61 or higher is used as a provisional diagnosis of PCBD and PGD. The developers report excellent internal consistency (Cronbach's alpha = .95) and evidence of concurrent validity through correlations with measures of psychopathology and quality of life. Higher scores among those bereaved by violent and unnatural causes and among those with multiple losses support the measure's construct validity. The measure was originally developed in Dutch; the wording of some items was slightly altered from the developers' translation to suit an English-speaking audience (e.g., “I had trouble to accept the loss” was changed to “I have trouble accepting my feelings about the loss”).

Seven related items were added asking about coping with difficult grief-related experiences rather than the severity of the experiences themselves. These items were matched to TGI-SR questions (e.g. “I feel bitterness or anger related to his/her death” was followed by “I can cope with my bitterness or anger related to his/her death”) and then summed separately to create an overall coping score, with higher scores indicating better coping.

**Qualitative interviews.** Semi-structured interviews were used to develop a fuller understanding of the opportunities and challenges associated with utilizing care farming as a therapeutic intervention for people experiencing grief and bereavement. Interviews were conducted by a member of the research team who was not involved in the delivery of the intervention. Interviews lasted for an hour, on average, and were conducted via phone or online video-call. Evidence suggests that qualitative telephone interviews can limit emotional distress because of the comfort experienced through virtual communication (Mealer and Jones, 2014). Open-ended interview questions were asked, including, “What attracted you to the care farm?,” “Did you learn anything about your grief during your time at the carefarm?,” and “Has your time on the farm changed anything about how you live your life?” The semi-structured nature allowed participants to focus on areas they felt were most important about their subjective experiences at the care farm. Interviews were recorded with participants' consent and transcribed. Transcripts where then analysed using NVIVO, following a ‘thematic analysis’ approach – ‘a method for identifying, analysing and reporting patterns (themes) within data’ (Braun and Clarke, 2006: 79). All participants have been ascribed pseudonyms.

## 6 Results

### 6.1 Demographics

The quantitative sample consisted of 22 bereaved individuals, three couples and 16 who came as individuals. The sample was 68.2% female, predominantly of White European descent (77.3%), and with a mean age of 42.41 (*SD* = 11.37) years. Religiously, most identified as Christian (36.4%) or agnostic or atheist (27.2%). Most participants were married or partnered (63.6%) and employed (81.8%). The most frequently selected categories for annual family income were $75,000 to $100,000 (31.8%) and $50,000 to $75,000 (36.4%).

Most participants had experienced the death of a child (77.3%) and the remainder had experienced the death of a sibling (22.7%). Most reported that the death had been sudden and unexpected (86.4%), while others reported that it was the result of a long-term illness (4.5%) or marked “other” and indicated that the death was from a terminal illness within a year of diagnosis (9.1%). The mean time since the loss was 3.39 (*SD* = 4.36) years. The age range of the loved one at the time of death was most frequently before/during birth to three years (36.4%) and 21 years and older (36.4%). The majority (72.7%) reported having spent time previously on a farm and around farm animals; 40.9% reported they were taking psychotropic medications at pretest. When asked to rate how valuable the carefarm experience was at posttest, 95.5% chose “extremely valuable” and 4.5% chose “valuable.” Demographic and loss-related data are provided in Table 1 (see Table 2).

**Table 1:** Demographic and Loss Descriptive Statistics (n = 22).

Demographics	%	Mean (*SD*)
Female	68.2	
Age		42.41 (11.37)
*Ethnicity*		
European descent	77.3	
Other	22.7	
*Religion*		
Christian	36.4	
Agnostic or atheist	27.2	
Spiritual, not religious	18.2	
Other	28.1	
*Marital status*		
Married/partnered	63.6	
Single	22.7	
Divorced	13.6	
Employed (full or part-time)	81.8	
*Annual income*		
<25k	9.1	
25k-50k	–	
50k-75k	36.4	
75k-100k	31.8%	
100k-150k	9.1	
>150	13.6	
**Loss information**		
*Relationship to deceased*		
Parent	77.3	
Sibling	22.7	
Sudden/unexpected death	86.4	
Years since loss		3.39 (4.36)
Age of loved one at death		
Birth to 3 years	36.4	
3–11 years	9.1	
12–21 years	18.2	
21 + years	36.4	
**Other**		
Psychotropic medication use	40.9	
**Carefarm experience**		
Valuable	4.5	
Extremely valuable	95.5	
Prior time with animals	72.7

**Table 2:** *TGI-SR and Coping Scores*.

Subgroup	TGI-SR pre	TGI-SR post	Coping pre	Coping post
	*Mean (SD)*	*Mean (SD)*	*Mean (SD)*	*Mean (SD)*
Entire sample	64.09 (11.08)	50.09 (8.89)	22.05 (4.35)	25.91 (3.90)
*Parents (n* = *17)*	62.76 (10.58)	51.29 (9.21)	21.76 (4.84)	25.29 (4.22)
Child < 3 years (*n* = 8)	59.63 (7.76)	48.86 (7.92)	23.25 (3.28)	26.50 (3.07)
Child 3–11 years (*n* = 2)	53.50 (10.61)	47.00 (2.83)	26.50 (.71)	30.00 (.00)
Child 12–21 years (*n* = 3)	75.33 (6.81)	59.00 (15.72)	20.33 (5.86)	24.00 (3.46)
Child >21 years (*n* = 4)	64.25 (11.98)	52.50 (7.05)	17.50 (5.45)	21.50 (5.07)
*Siblings* (*n* = 5)	68.60 (12.80)	46.00 (6.93)	23.00 (2.00)	28.00 (1.22)

### 6.2 Traumatic Grief inventory-self report results

Cronbach's alpha, which is a measure of a scale's internal consistency, or the degree to which scale items measure the same construct (Tavakol and Dennick, 2011), was good at pretest (0.83) and adequate at posttest (0.77) for the TGI-SR. The mean TGI-SR score was 64.09 (*SD* = 11.08) at pretest and 50.09 (*SD* = 8.89) at posttest. The scores at both times were normally distributed, with skewness and kurtosis values under 1, and had no outliers. Paired-samples *t*-tests showed a significant reduction in scores from pretest to posttest, *t*(21) = 5.57, *p* < .0001, 95% CI [8.77, 19.23]. Initially, 50% of scores were above the clinical cutoff of 61. At posttest, this figure had fallen to 18.2%.

The internal consistency for the coping scales created to complement the TGI-SR was low as measured with Cronbach's alpha (0.58 at pretest, 0.60 at posttest). These distributions approached normal but had skewness values of −1.15 at both times and kurtosis values of 1.26 at pretest and 1.74 at posttest. There was one outlier in the posttest coping distribution, on the low end. The mean pretest coping score was 22.05 (*SD* = 4.35) and the mean posttest score was 25.91 (*SD* = 3.90). Paired-samples *t-*tests showed a statistically significant increase in coping from pretest to posttest, *t*(21) = −5.86, *p* < .0001, 95% CI [-5.23, −2.49].

Because time since the loss is often thought to impact the intensity of grieving, a univariate regression analysis was run to determine whether this was true in the current sample. Time since the loss was not a significant predictor of TGI-SR score at pretest, *F*(1, 20) = .05, *p* = .82, or at posttest, *F*(1, 20) = .35, *p* = .56.

The mean TGI-SR and coping scores were broken down according to whether participants had experienced the death of a child or sibling. Within the child loss group, mean scores were further broken down according to the age of the child at death. Bereaved siblings (n = 5) experienced a higher mean reduction in TGI-SR scores from pretest to posttest (22.6) than did bereaved parents (11.34).

### 6.3 Qualitative results

Of the 22 participants who completed the TGI-SR, 21 were interviewed (one individual did not participate). Across the corpus of interview data, three themes stood out as particularly important when participants reflected upon their experiences at the carefarm and how it affected their grief. Firstly, the “restorative spaces” of the carefarm: the landscape, atmospheres, and natural milieu of the farm, and how people interpreted and interacted with these elements, in metaphorical, embodied, and sensuous ways. Secondly, the “community” of the carefarm: being in a place where grief is accepted and depathologized, with others who could empathise and relate, and where it was acceptable to talk about grief and trauma. Thirdly, the connection to animals: both the animals as a metaphor and signifier that instilled hope, but also the opportunity to develop empathy and compassion through encounters and relationships with the animals. Particularly, it was the ability to do all of this in parallel with the focused counseling, the animals, nature, and a sense of community creating beneficial opportunities for contemplation, reflection, and depressurisation that enhanced therapeutic processes.

#### 6.3.1 Restorative spaces of the carefarm

When reflecting on their experiences of the carefarm, an important theme for many participants was the place of the carefarm itself, its material and symbolic geographies and location within “nature.” The integration of the formal aspects of counseling with the outdoor environment filled with animal life helped many participants feel more comfortable discussing sensitive topics, particularly when contrasted with more traditional forms of psychotherapy.

“I've been through regular counseling before where you sit in a room. It's just so different when you're outside. I guess it's just enough of a distraction to let you relax a little bit. So I'd be able to open up a little bit more, it just helps you, it helps me relax and feel more comfortable and open.” – Anna

*“*Thing I liked is that it was not clinical. It didn't feel like you were in any kind of facility.*”* - Joe

This idea of the landscape of the carefarm enabling people to “open up” and “feel safe” was a frequent theme:

“You just feel like you're in a very safe place and it's like nobody needs to tell you to feel that way. You just, you just do. I can't describe like how that happens […] it's just like a safe haven and like a safe place and it just makes it, it changes the dynamic of how you feel while you're there.” - Jason

“I say the word safe, that's really important, right? For me that was huge. It's safe. It feels safe. If you're going through bereavement, it's so important that you have that there, that that is available. I wasn't expecting that I would get all those emotions, and how strong they were. But because I was in that environment, it was okay.” - Alice

The “affective atmosphere” (Anderson, 2009) of safety that participants associated with the space of the carefarm shaped how they interacted with the formal counseling. Participants felt enabled to explore their emotions and perspectives surrounding trauma and loss. For some, this sense of safety emerged from the presence of, and relationships with, nature that the carefarm curated:

“I think part of creating that really safe space, where even the animals were on the same footing as the humans, was just respect for nature, whether it was plant or animal or human and I think that was actually really important.” – Nicole

“The care farm, like just being in wandering around and touching the animals and being with animals. And it was we were so receptive and comfortable because we were so at ease from just existing in a world where it wasn't like, come into my office, sit down.” - Daniel

“And of course the nature around you, lends to that as well, the little river, the little creek that goes by, the beautiful trees, the flowers, you know, all of that adds to it as well and it just feels like a safe place. And when you go through a trauma, like what I've been through, what you're really seeking more than anything else is safety. Cause you don't feel safe anymore. And so that provides a safe place.” - Jennifer

The natural environment of the farm was discussed regularly by participants, particularly though the lens of grief and bereavement:

“There's just so much beauty in nature. It just feels so life-sustaining, it's a nice place to be when you're not feeling, you know, when you're feeling down about anything but especially something like this […] just having beauty, you know, when you've seen something ugly, just beauty is good.” – Melissa

“I was really attracted to the water and how like, you know, the water can be such a powerful metaphor too for like, especially like when dealing with grief.” - Eric

Being in, and with, nature on the carefarm as part of the therapeutic intervention, appeared to benefit participants and generate a sense of comfort. Some found useful metaphors from being outdoors during therapy sessions. Perhaps importantly, these metaphors were ones that participants located themselves, used to (re)narrate their ongoing relationship and ways of coping with grief:

“I saw this grapevine, and the grapevine was being supported by the fence, and eventually that fence would be taken away and then all the grapevines were sort of holding on to each other, that metaphor really helped me in thinking about my support network, so it was things like that, whereas before I'd have gone ‘oh it's just a grapevine you know.'” - Nicole

Others derived benefit from an embodied and sensuous encounter with the carefarm's naturescapes. These were invoked by participants through memories and material collected at the farm to provide a link back to, and ongoing engagement with, the therapeutic processes initiated at the farm:

“It's also a sensory space, but the smells and visual stimulation and I think that's really important to have, to be able to feel that way in different spots of the farm. It's just in a very special sensory spot. So I can remember like certain smells or feelings and then certain areas. […] I gathered a bunch of like sand and silt and some of the vegetation from sticks, a little bit of bamboo and I gathered that in a jar and so I have that here. That's a reminder of the good times. And the sad times, but you have to embrace every single angle. I think that's important.” - Eric

A sense of health and a sense of place are deeply and dynamically intertwined (Gastaldo et al., 2004). Participants found meaningful experiences within the therapeutic landscape of the carefarm itself. The setting opened up opportunities for them to feel safe to engage and explore ongoing psychological distress, as well as offering the chance for curated, yet individualised, meaningful encounters with aspects of nature.

#### 6.3.2 The community of the carefarm

Participants frequently reported that being part of a community of people affected by grief was a beneficial facet of their carefarm experience, countering the loneliness and social isolation common in bereavement (Schwab, 1996):

“I have felt very alone and isolated and that there's something wrong with me and this isn't okay. And so I'm going to say from going to the carefarm, I have felt better actually. I felt significantly better.” - Alice

“I was nervous coming to the carefarm because I didn't know what to expect. I was definitely stepping out of my comfort zone. But I was met with such compassion and a complete understanding of where I was, it wasn't something I had to explain, you know. It felt very, very nice to be so understood.” - Sophia

This sense of understanding, derived from the formation of a community fellowship of grief, was crucial for many participants in enabling them to feel safe and able to engage with the intervention itself. Indeed, many linked this sense of community to the idea of personalised care:

“I could not find anyone around here who really understood. I've been to three therapists now, and you know, they just, I feel like they don't quite get it.” – Laura

“We felt validated and we also felt like we could tell anything and it'd be okay.” - Daniel

“It's getting out there and going to a place specific to cope with grief is what really attracted me to that.” - Anna

The community norms, like the acceptability to talk about grief and traumatic loss, were particularly important in creating a place where people felt comfortable and accepted when discussing topics normally stigmatised by society:

“It's not always comfortable to talk about, you know, not everyone wants to hear about your dead baby all the time. And it just felt like a place that was comfortable and welcoming and you know, designed to help people, designed to help us.” - Andrea

Participants identified not just the ability to talk, but also the ability to be heard and understood as an important aspect of their experience. The fact that the community consisted of others who could both empathise and relate was regarded by participants as crucial:

“That I can speak to this person and they actually understand what I'm saying as opposed to just hearing what I'm saying. […] So yes, coming to the carefarm is the first time for us that we didn't feel alienated from reality and the rest of the world.” - Amanda

The opportunity to meet other people affected by traumatic grief, and participating in a community, was felt to be beneficial. This, again, acted to establish the carefarm as a safe place, where discussing sensitive topics normally outside the purview of ordinary conversation was normalised:

*“*So that was a new experience for me, being with people in person that have also lost siblings and being able to talk to them about it. […]That was the first part of realizing, you know, I'm not alone in this, you know, other people have been here in this horrible pain.” *- Katy*

Community members supported each other in sharing and learning ways of being-with journeys in grief:

*“*They helped me make those connections, and also to not feel bad about feeling quite good, alongside feeling absolutely distraught. So that sort of impermanence of feeling. That it comes and it goes, and one minute you might be crying on the floor, and the next minute you might feel like going for a run, and just, allowing that, and for it to be okay. Lots of grief books just talk about how awful your life is, and how you're not able to breathe every minute, and I was like, it's like that sometimes, but not all the time, and yeah, they validated that sense in me, that feeling.” - Nicole

“Prior to coming to the carefarm I was so worried I was doing grief wrong.” - Sophia

For some, the community validated their feelings. Many participants discussed fear around “doing grief the right way” or grieving “properly” that were assuaged through conversations with others. Contact with other grievers at the carefarm, and the pooling together of lived experiences, as opposed to more medicalised framings of grief, empowered participants in the legitimacy of their own emotional and affective states.

#### 6.3.3 Connection to animals

The carefarm's animals, who had all been rescued from abuse or neglect, and the sense of connection to them, were significant for participants. For some, the presence of the animals acted as an important initiating factor in visiting the carefarm for support, with the animals signifying the philosophy of care built in to the intervention:

“I don't think I would've gone out there if there had not been animals. I think it would've, I think I would've just passed right over it as just another place.” - Katy

“I need to be like treated gently and I'm not always getting that in the world even though people try, but I'm not always getting that. And I felt like a place that helped that, you know, has rescue animals and that it would be like that.” - Laura

Critically, having animals present acted to create space for people to process the formal counseling aspects of the intervention:

“Anything you learn in the counseling, you can take it to the animals and they help you kind of digest it.” - Jennifer

“I feel like the two parts are, you know, going and talking is a very intensive part where you have to dig deep and experience a lot of intense emotions and things like that. But then you get to go out and have the second part, which is you can decompress and just kind of process by walking around and being with the animals and going and being with an animal who is completely silent for the most part, but knows what it is to suffer.” - Amanda

Spending time with the animals encouraged reflection and processing, an outlet for solitude, and meaningful diversionary tasks. Particularly important was the carefarm's focus on caring for animals rescued from abuse and neglect. Participants discussed feeling a strong sense of connection, empathy, and relationship with the animals' traumatic histories:

“Connecting with them on the same level, you know, they too have gone through a traumatic experience and being able to connect with them, and share that.” - Anna

Gorman (2019) has described how animals’ biographies can play a role in how people engage with, and come to benefit from, care farming practices. Recognizing familiarity in other beings can enable empathy and critical engagement (Hayward, 2012). For participants who visited the carefarm, the animals emerged as a model and symbol of hope and ongoing resilience in the face of trauma:

“I think there is something very powerful in the fact that they're thriving and alive, and some of them spunky, just living this existence. Just that they triumphed over a level of trauma and grief of their own. And that kind of provides an example of something that is possible to achieve even regardless of how bad things might appear or might be in a moment. So it just kind of gives you that hope, I guess, that things can be different.” - Melissa

*“*I think that that definitely makes a huge impact as well. I mean, just knowing that those animals have been through hard times just the same and you can see it in their eyes, there's definitely a connection there*.”* - Jason

“I was sitting there interacting with them and thinking how is this helping us? Cause it does feel like it's helping us. And I think, having processed it now, I think it's interacting with animals that have been traumatized and seeing that they are not the same as non-traumatized animals. There's a difference. They are more skittish and you have to really give them more time to trust you. But, they are able to still feel love. And I mean, they're able to thrive and live in this beautiful place. And I think that we hadn't really recognized that this traumatic life was forever. You just think like, oh, I'm gonna feel better in a week or six months or whatever. And I think that sort of helped me move in the direction of recognizing that we are forever changed by this event and that that's okay. We didn't choose it, but we're going to be okay. The lives that these animals lead are full of love and beauty and they didn't choose either, and they're doing okay.”

- Andrea

Participants regularly recounted their encounters with animals as a meaningful and important part of the intervention. In the same way that Rose has argued that “certain kinds of landscape, encountered simultaneously as both natural, objective realities but also ‘representations’, can ‘mirror’ emotional states back to the viewer, precipitating a beneficial mentalising process” (2012, p. 1383), so too did the carefarm's animals.

## 7 Discussion

Both the quantitative and qualitative findings suggest that the carefarm intervention was experienced as beneficial by bereaved participants – becoming something of both a ‘therapeutic landscape’ and a ‘therapeutic community’. The mean pretest TGI-SR score of 64.09 was above the cutoff of 61 and was higher than the mean score of 53.41 in a sample bereaved by unnatural or violence causes and the mean score of 49.36 in a sample with multiple losses (Boelen and Smid, 2017). These scores, along with participants' narratives, suggest a high level of bereavement-related distress prior to the carefarm intervention.

A significant reduction in grief intensity on the TGI-SR was observed following the carefarm intervention, with a mean improvement of 14 points. At posttest the mean score had fallen below the clinical cutoff. The reduction in the percentage of participant scores above the clinical cutoff from 50% at pretest to 18.2% at posttest further underscores the improvement observed in this sample. Interestingly, the bereaved sibling group started out more highly distressed and experienced greater improvement than the bereaved parent group. The bereaved sibling group comprised a very small sample and the reason for this difference requires further study.

Although the analysis revealed a significant increase in coping with grief-related distress, the newly created scale used to measure this showed poor internal consistency, with Cronbach's alpha values of .58 at pretest and .60 at posttest. The range for Cronbach's alpha encompasses 0 to 1, and values below 0.70 are generally considered unacceptable (Tavakol and Dennick, 2011). Scales with few items typically have lower alpha values, so it is possible that a longer version with more than the seven items included here would show stronger internal consistency; alternatively, it is possible that this scale included heterogenous constructs instead of measuring a single construct, which would result in a lower alpha value (Tavakol and Dennick, 2011). Because of the low alpha values and this study's inability to explain why these occurred, no strong conclusions, other than a report of participants subjective experiences of coping, related to the intervention's impact on these coping-related areas can be drawn at this time.

The themes identified in the qualitative analysis begin to explain the quantitative data, highlighting the value that a mixed methods approach can bring to understanding the complexities of bereavement care. The qualitative results suggest that a reduction in grief intensity can be realised through the creation of tailored, nuanced, and personalised care in bereavement support. Indeed, the qualitative results suggest that this framing can influence individuals engaging with therapeutic processes to begin with. It reiterates Hall's argument that “interventions must be tailored to the uniqueness of the person, relationship and circumstances that characterise a client at a particular point in time as they grieve a specific loss” (2014, p. 12).

The opportunity to share freely, and be met with empathy and understanding, was of a critical importance. Many participants described previous negative experiences of “catch-all” services for mental health. As Butler describes, when discussing grief, “we are talking about affective responses that are highly regulated by regimes of power and sometimes subject to explicit censorship” (2009, p. 39). The carefarm, co-constituted by a “therapeutic community,” acted as a place where social conventions were cast off and participants were free to openly explore and process emotional traumas. Similarly, Butler (2016) reminds us that grief has a geography, and that there are norms not only of when – but also of *where* – a life is grievable. Visiting a particularized place where grief is normalised and depathologized can allow for the emergence of productive and beneficial encounters, free from societal conventions and stigmas.

The results from this evaluation reflect the benefits of creating specific communities and spaces for those affected by grief to find solidarity, support, and resilience. It also suggests the value that a ‘therapeutic landscapes’ approach might bring to thinking about bereavement support. This is an area where there has been little cross-fertilisation, and we would be keen to see others working at the interdisciplinary intersections of grief and health geography further interrogate the benefits on offer here. Indeed, if it is not possible to separate experiences of health and wellbeing from the places in which they are experienced (Kearns, 1993), then this is a vital lesson for thinking about the ‘where’ of bereavement care, and the (un)therapeutic landscapes that constitute services for the bereaved. Many of our participants reflected on the possibilities evoked by the place of the carefarm, in contrast to that of the more traditional therapeutic landscape of counseling offices. Back in 1992, Gesler (744) argued that thinking about healthcare requires recognizing how the “social and spatial are intimately intertwined”, and our work here, reiterates that within the context of bereavement care. However, and importantly, it is also a ‘social’ that is actively co-constituted by animals too, and greater efforts are still needed to integrate discussions of non-human agency into theoretical deployments of the therapeutic landscape framework.

Regarding the matter of animals, specifically, Marr et al. (2000) previously found, incorporating animals into therapeutic processes can play an important role in enhancing the benefits and improving the effectiveness of conventional therapy. In the current study, the presence of animals, and other aspects of nature, acted as “enabling resources” that afforded, empowered, and made possible other therapeutic activities (Duff, 2011). Schneider and Harley found that the presence of animals within therapy “enhances perceptions of therapists and the willingness to disclose to therapists” (2006, p.136). Again, this was echoed by participants, who contrasted positive carefarm experiences with previous negative experiences when attempting to engage with counseling, therapy, and bereavement support. The carefarm animals acted as an important signifier of the possibility of different forms of relationships with therapists and therapy.

From a physiological perspective, hands-on encounters with animals can significantly lower physiological markers of stress, such as cortisol, heart rate, and blood pressure (Beetz et al., 2012).^2^ The incorporation of these benefits into therapy and support hold great potential for grieving individuals. Importantly, animal-assisted-therapies are frequently viewed as more acceptable forms of treatment than medication in the context of mental health (Rabbit et al., 2014). Given the lack of consensus on the use psychotropic medications for grief (Thieleman and Cacciatore, 2014), there are opportunities to develop forms of grief support that allow people to access care that resonates with them, rather than being limited to a medical model.

Importantly, it was the involvement of animals who participants identified as having also experienced trauma that enabled the production of what was experienced as a therapeutic space and therapeutic community (of more-than-human members). This finding suggests that grieving individuals might not benefit to the same extent from more conventional care farms, where the incorporation of livestock that are ultimately destined for slaughter could be a source of emotional stress for participants (Gorman, 2017a).

These results add to the growing body of evidence on care farming in general (Ellingsen-Dalskau et al., 2016; Hine et al., 2008; Iancu et al., 2014; Kogstad et al., 2014; Leck et al., 2015; Pedersen et al., 2012) and offer the first empirical evaluation of its benefits related to traumatic grief. Although the quantitative results show a decrease in grief intensity after the intervention, this study is limited by the lack of a comparison group that would strengthen the validity of findings. Additionally, results from both the quantitative and qualitative components cannot be generalized beyond this sample. This study relied on a self-selected sample of individuals who may be very different from other groups of grieving individuals. It is likely that a care farm experience will not be comfortable or acceptable to all individuals. However, for those who expressed interest and comfort, the experience was viewed as valuable and beneficial. Despite these limitations, this study's results are encouraging and seem to warrant further investigation into the outcomes and possibilities that incorporating animals into therapeutic processes, spaces, and communities might have for those affected by grief and bereavement.

## 8 Conclusion

This study's findings support the idea of traumatic bereavement as a complex experience affecting individuals in multiple domains, one that may require skilful and holistic community-based interventions to promote emotional and physical adjustment and well-being. Hinchliffe et al. have recently argued that making health possible “requires not just investment in biomedical remedies […] but also recognition of the relational, cultural and environmental – that is, nonpharmacological – factors that enable people to cope with life crises and transitions” (2018, p. 8). This is a perspective that has particular resonance in designing support services for the traumatically bereaved. Drawing on the investigations here, the focus of efforts should lie in creating therapeutic landscapes and communities that enable sensitive and egalitarian encounters with people, place, and nature in ways that help those affected by traumatic grief.

Carefarming, as presented here, is one example of a pathway toward therapeutic benefit for this population. For participants, this therapeutic landscape came to be understood and experienced as a restorative space and community. Interspersing focused counseling with encounters with animals and nature appeared to enhance engagement with more formal narrative therapies, as well as lead to significant reductions in distressing reactions to their own grief. Beneficial opportunities for contemplation, reflection, and depressurisation were enabled by emplacing support within the specific social and spatial contexts of the farm.

This is the first empirical investigation of the effects that care farming can have on individuals affected by traumatic grief. There are important implications here for the further uptake of this style of intervention as means of supporting grieving individuals and families. Although these preliminary findings are encouraging, there are further questions to be asked, and more research that expands the evidence base for green care, both in general, and in its application to trauma and grief, is needed. Specifically, we would encourage more longitudinal studies of carefarming, more interdisciplinary investigations into both carefarming and traumatic grief, and more work that seeks to clarify the potentials, and moral complexities, of integrating animals into the delivery of healthcare interventions.

## Funding

This study was funded in part through an internal grant from 10.13039/100007482Arizona State University’s Watts College of Public Service and Community Solutions. Additionally, RG's time on this study was enabled by the Wellcome Trust collaborative award on the Animal Research Nexus (205393/Z/16/Z).

## Footnotes

1 Whilst the literature commonly used the phrase ‘care farms’, the terminology preferred by the intervention site involves eliminating the space between the adjective and the noun, using “carefarm” together as a statement about their commitment to caring for both humans and animals together.

2 However, the majority of physiological evidence at present is based around encounters with dogs (with a small literature considering equine-therapy (Pendry et al., 2014)). Further expanding the species studied for their therapeutic and stress-relieving benefits will be an important future direction for research.
